# Collaborative decision-making for localized emergency response in major railroad projects

**DOI:** 10.1038/s41598-025-09190-w

**Published:** 2025-07-21

**Authors:** Yuanyuan Lei, Jingxiao Zhang, Ruixue Zhang, Lean Yu, Pablo Ballesteros-Pérez, Martin Skitmore

**Affiliations:** 1 School of Economics and Management, Chang’an University, Xi’an, China; 2 School of Economics and Management, Xi’an Shiyou University, Xi’an, China; 3 Business School, Sichuan University, Chengdu, China; 4 Innovation and Sustainability Research Centre (PRINS), Universitat Politècnica de València, Valencia, Spain; 5 Faculty of Society and Design, Bond University, Gold Coast, Australia

**Keywords:** Evolutionary game, Major railroad projects, Localized emergency response, Multi-agent decision, Environmental social sciences, Natural hazards, Engineering

## Abstract

This paper examines major railroad projects’ emergency response decision-making process, focusing on timeliness, cost-effectiveness, and public involvement. It explores the optimal collaborative strategies of local governments, enterprises, and the public during the construction phase. A novel tripartite decision-making framework is proposed, based on an asymmetric evolutionary game model and dynamic simulation methods. The results show a stable equilibrium in emergency response strategies emerges when public engagement is prioritized. Government subsidy policies are found to significantly influence the strategic choices of all agents, emphasizing the critical role of coordinated collaboration. The study highlights the importance of public participation and multi-agent cooperation in addressing complex emergency challenges. These findings provide valuable guidance for improving localized emergency response strategies, enhancing collaborative decision-making, optimizing resource allocation, increasing response efficiency, and supporting policy formulation in major railroad projects.

## Introduction

As a key component of China’s infrastructure and national economic operations, major railroad projects in complex and hazardous areas have become a strategic priority in recent years^1^. These projects face considerable challenges due to high altitudes, seismic activity, and other adverse geological conditions^2^. Since 2008, China has intensified the construction of railroad networks in the western region, including the Sichuan-Tibetan Railroad and the Sichuan-Qinghai Railroad on the Qinghai-Tibetan Plateau^3^. The harsh natural environment in these regions complicates emergency response efforts, necessitating multi-level government collaboration, coordination with rescue agencies, stringent time constraints, and advanced materials and technologies^4^. As a result, achieving a balanced emergency response that considers timeliness, cost-effectiveness, and public involvement is important for both the construction and stable operation of major railroad projects.

The complexity of emergency responses in such projects arises from challenging terrain, unpredictable hazards, and the requirement for rapid and coordinated action. Effective coordination across multiple levels of government, enterprises, and public agencies adds further difficulty. Disasters such as earthquakes, landslides, and floods introduce dynamic and uncertain conditions that demand immediate, flexible responses. Additionally, decision-making needs to balance the differing interests and capacities of local governments, enterprises, and the public. Constraints such as financial resources, time pressures, and safety concerns make emergency response a multifaceted and high-stakes task. Managing emergencies effectively in this context requires rigorous planning, real-time coordination, and adaptability.

Previous research into localized emergency response decision-making for railroad projects has largely concentrated on ambulance vehicle allocation^5^, emergency resource scheduling under uncertainty, facility siting, route optimization^6^, and disaster prevention communication system bandwidth in disaster prevention^7^. However, there is limited focus on the evolution of scenarios over time within emergency decision-making processes. Recent studies have further advanced the understanding of multi-agency cooperative decision-making in dynamic emergency environments. Effective communication and resource sharing are crucial for reducing response times in large-scale disaster scenarios^8^. Shared identity among emergency responders from different agencies significantly enhances interoperability and coordination during multi-agent emergency response exercises^9^. A dynamic optimization model enables real-time strategy adjustments in multi-agent emergency responses to evolving disaster conditions^10^. Based on the TOPSIS decision support tool, it was found that improving the leadership skills of emergency managers was the optimal intervention in multi-agency emergency response activities, followed by promoting community participation in decision-making and response activities^11^. These findings highlight a gap in current research: the need for a comprehensive framework to address the evolving dynamics of emergency response in major railroad construction projects.

Collaborative decision-making integrates expertise from diverse stakeholders to enhance decision accuracy. This approach has been widely studied in emergency management, supply chain systems, and transportation^12^. The focus has largely been on optimizing response time and resource use, with less attention given to the quality and influencing factors of collaboration^13^. Studies have shown that collaborative decision-making positively impacts emergency response outcomes^14^, and its quality has become a benchmark for evaluating emergency systems’ performance^15^. In railroad projects involving stakeholders such as local governments, enterprises, and the public, collaborative processes encounter obstacles including cross-regional coordination and the need to establish trust in high-risk scenarios. These challenges remain insufficiently explored, highlighting the necessity of tailored collaborative models.

Railroad construction is often constrained by geography, climate, and environmental regulations. Sites are typically located far from urban centers, where access to emergency supplies and equipment is limited, and landslides or mudslides hinder transportation. Timeliness is therefore a critical factor. Localized emergency response systems offer advantages, such as resource availability, faster response, local community support, and better adaptability to environmental conditions. These systems also benefit from local governments’ familiarity with the terrain and available resources.

Nevertheless, railroad projects involve cross-regional dimensions. Effective response strategies need to integrate localized strengths with regional resource coordination. In some areas of China, recurrent disasters have prompted the government to increase subsidies for local disaster relief organizations. However, these trial measures focus predominantly on post-disaster recovery and lack systematic consideration of strategic decision-making by local agents or the role of government during the emergency phase.

Evolutionary game theory (EGT) offers a robust framework to analyze the complex interactions and time-dependent evolution of strategies in emergency management. Traditional game theory has primarily been used to model emergency responses through static frameworks. These approaches, while foundational, assume fixed strategies and rational behavior, making them less suitable for the dynamic and uncertain conditions of real-world emergencies^16^. For instance, classical models such as the Nash equilibrium assume that agents have perfect rationality and complete information^17^. Time pressure and uncertainty render these assumptions invalid in emergencies, especially in railroad projects. Behavioral game theory and mechanism design provide alternative frameworks by introducing bounded rationality and incentive mechanisms suitable for complex environments^18^. EGT, originally proposed by Smith and Price (1973) and further developed by Nowak (2006), models the adaptive evolution of strategies based on agents’ success or failure in previous interactions^19^. This capability makes EGT highly suitable for modeling the behavior of multiple agents involved in emergency responses, where collaboration and conflict are shaped by continuous feedback and external uncertainties such as public opinion, policy shifts, or disaster intensity. EGT enables simulation of the strategy evolution of agents, making it a relevant tool for understanding how decisions adapt over time to changing conditions. In the context of major railroad projects, EGT facilitates analysis of the collaborative dynamics of local governments, enterprises, and the public. The model helps identify long-term equilibrium strategies and evaluates how emergency responses and government subsidies influence strategic behavior, ultimately improving emergency management.

This study contributes in two key ways. First, it aligns with the principles of China’s emergency management system, particularly the “hierarchical responsibility and territorial management” model, and the specific requirements of major railroad projects. It proposes local governments as primary agents in interdepartmental collaboration. Through an asymmetric evolutionary game model, it dynamically analyzes cooperative emergency decision-making involving governments, enterprises, and the public. This approach encourages integration between emergency management and project construction management. Second, a dynamic evolutionary game model is introduced that accounts for limited resources, the urgency of rescue, and public interest. Three strategic scenarios are designed: economic, timeliness-focused, and coordination-based. These scenarios assess how government investment, economic outcomes, and public opinion influence localized emergency response strategies. The findings offer scientific guidance for improving emergency response effectiveness in major railroad projects.

The remainder of this paper is structured as follows. Section "Literature review" reviews the relevant literature. Section "Collaborative decision-making evolutionary game" develops a tripartite evolutionary game model involving three strategies and considers strategic consistency. Section "Evolutionary stability strategy analysis" analyzes equilibrium stability under tripartite collaboration and explores stable strategies in dynamic contexts. Section "Simulation results with three scenarios" presents numerical simulations illustrating the impact of stochastic disturbances on stakeholder strategies. Section "Discussion" offers further discussion, a summary of key insights, and identification of research gaps. Section "Conclusion" concludes the paper and outlines directions for future research.

## Literature review

The literature demonstrates that research into localized emergency responses for major railroad construction projects has primarily focused on resource allocation and logistical support. However, limited attention has been given to the characteristics of multi-agent cooperative decision-making in dynamic and multi-factorial emergency scenarios. In particular, the interplay between different emergency response strategies and government subsidies remains insufficiently explored. Furthermore, previous studies have not adequately examined the role of time-dependent evolution in shaping emergency response decisions.

In the context of localized emergency responses for major construction projects, establishing regional emergency response systems and ensuring stable, reliable, and timely decision-making are critical for effective outcomes and loss reduction^20,21^. Several studies have explored advanced modeling approaches to optimize emergency response systems to improve response times and outcomes^22^. By integrating best practices from emergency management in natural disasters and major accidents^23^, local governments can revise disaster response plans and improve their effectiveness through both internal motivation and external policy incentives^24^. This process supports the development of management frameworks characterized by safety prioritization, structural integration, stakeholder diversity, and flexible adaptation of emergency plans^25^. Moreover, major railroad emergency responses’ time constraints and scheduling pressures necessitate cooperation across multiple agents, dimensions, and levels^26^.

The effectiveness of emergency response in complex scenarios depends heavily on high-quality collaborative decision-making among multiple stakeholders^27^. Academic research has emphasized multi-agent game models involving governments and social organizations, specifically focusing on public opinion transmission mechanisms between the public and government departments^28^. Successful emergency responses rely on inter-organizational collaboration, communication, and adaptive capabilities to ensure system viability and efficiency^29^. Previous studies also developed emergency materials storage models to analyze government-enterprise reserve decisions^30^, and proposed optimal regional material reserve strategies through government-private sector cooperation^31^. System optimization has been widely recognized as a means to enhance emergency management capacity^32^.

In terms of evolutionary game applications in emergency response decision-making, prior studies have examined local government organisations’ behavioral evolution and strategy stability during disaster relief operations^33^. Tripartite evolutionary game models have also been used to explore the dynamic evolution of evacuation decisions and joint emergency material reserve strategies involving governments, enterprises, and the public^34^. Beyond the context of railroad emergency response in China, EGT has been applied in international studies such as implementing the European Railway Traffic Management System^35^ and analyzing chlorine rail transportation safety in the Texas-Illinois corridor in the United States^36^. These studies have highlighted the strengths of game-theoretic approaches in modeling stakeholder interactions under complex and evolving conditions.

Nonetheless, gaps remain in the literature. Specifically, previous research did not fully address the multi-agent cooperative decision-making characteristics of emergency responses in major railroad construction projects, particularly under dynamic and multi-factorial conditions. Nor does it sufficiently analyze how different emergency strategies interact with government subsidies.

This study applies an evolutionary game model to investigate how key parameters influence the dynamic process of emergency collaboration. The objective is to offer theoretical guidance for developing emergency rescue planning mechanisms in major railway projects. The decision-making strategies of various agents involved in the emergency response process are systematically examined. The findings offer both theoretical and practical insights into improving emergency response mechanisms and enhancing rescue effectiveness.

### Collaborative decision-making evolutionary game

China’s railway emergency rescue mode gradually shifts to multi-agent collaboration^37^. Emergency rescue subjects’ cooperation strategies are influenced by horizontal organizational cooperation and vertical administrative constraints^38^. This paper illustrates the evolutionary path of the cooperation strategies of the government, enterprises, and public emergency organizations in emergency response.

In the context of localized emergency response in major railroad construction projects, the strategic cost choices of local governments, enterprises, and the public are usually the following:

#### Local government costs

- *Human resource input*: This includes the number of personnel from government functional departments; rescue personnel such as military and police, firefighters; and volunteers organized by the local government.
- *Material and equipment input*: The government procured and provided materials and equipment for emergency response.
- *Transportation and logistics costs*: These cover the transportation and logistics support provided by the government for rescue operations, including the cost of transporting people and supplies to the disaster area.

#### Enterprise costs

- *Human resource input*: The human resources invested by the enterprise in emergency response, including rescuers, professionals, and employees.
- *Material and equipment input*: The enterprise purchases and provides the materials and equipment for emergency response.
- *Maintenance and recovery costs*: The costs required for repairing affected facilities and resuming production and services, including equipment maintenance and building restoration.

#### Public relief costs

- *Individual contributions*: Funds are voluntarily donated by the public to relief agencies or disaster areas.
- *Volunteer labor*: Labor is voluntarily provided by the public, encompassing volunteers’ time and energy input.
- *Material donations*: The public voluntarily donates materials to relief agencies or disaster areas.

Analogously, in localized emergency responses to major railway engineering projects, the reputational impacts on local governments, enterprises, and the public are defined by specific indicators, such as the following:

#### Reputation indicators for local governments

- *Crisis management capability*: The efficiency and effectiveness in handling emergencies.
- *Rescue actions and response speed*: The promptness and effectiveness of rescue operations.

#### Reputation indicators for enterprises

- *Accident rate*: The frequency of accidents occurring within the enterprise’s operations.
- *Compliance with safety norms*: Adherence to safety regulations and operating procedures.
- *Coverage of safety training and education*: The extent to which safety training and education are provided to employees.
- *Response time to emergencies*: The swiftness of the enterprise’s response to emergencies.
- *Effectiveness of emergency response measures*: The efficacy of the measures taken during emergencies.
- *Collaboration and effectiveness of rescue operations*: The degree of cooperation and success.

#### Reputation indicators for the public

- *Public satisfaction with emergency response measures*: The public’s contentment with the measures taken during emergencies.
- *Opportunity and degree of public participation*: The extent and quality of public involvement in decision-making.
- *Public satisfaction with the feedback mechanism*: The public’s satisfaction with how their feedback is handled and addressed.

By defining these costs and reputation impacts, the collaborative decision evolutionary game model we propose aims to elucidate the dynamics and interactions among local governments, enterprises, and the public in localized emergency response scenarios for major railroad projects.

## Basic assumptions

The collaborative decision-making process during localized emergency responses in major railroad construction projects involves three key decision-making agents: local governments (A), enterprises (B), and the public (M). All three agents are assumed to be rational in their decision-making processes. Local government A is responsible for overall collaboration and resource allocation, while enterprise B includes railroad construction units and emergency material suppliers. Public M encompasses affected residents, experts, civil rescue organizations, and social media actors. During the emergency response process, different agents perceive and process information differently. However, how external information affects agents’ decision-making is important for improving the emergency response^39^. We assume two hypotheses.

### Hypothesis 1

*There are three pure strategies for the three agents of major railroad construction: an economic strategy, a timeliness strategy, and a coordinated strategy. Economic strategy focuses on controlling costs and achieving the greatest economic benefit. It includes cutting unnecessary spending, choosing the most cost-effective actions, and allocating resources based on careful cost–benefit analysis. The government may cut costs by streamlining staffing, improving material purchasing processes, and lowering administrative expenses. Timeliness strategy focuses on quick response and fast action. Its goal is to shorten response time by quickly mobilizing rescue teams, improving transport and logistics, and ensuring emergency resources reach disaster areas without delay. The strategy stresses speed and efficiency, reducing the time between decision-making and actual rescue. The government may invest more resources to speed up rescue operations. Coordination strategy emphasizes working together and sharing resources among government departments, businesses, and public organizations. The focus is on joint efforts between the government and other parties. The government boosts overall response efficiency by coordinating the resources and actions of all involved.*

The local government’s strategy set is S_A_ = {A1, A2, A3}, where A1 represents the economic strategy, A2 represents the timeliness strategy, A3 represents the coordinated strategy, and the local government probabilities of choosing these strategies are x(0 ≤ x ≤ 1), y(0 ≤ y ≤ 1), and 1–x–y, respectively.

The enterprise’s strategy set is S_B_ = {B1, B2, B3}, where B1 stands for the economic strategy, B2 stands for the timeliness strategy, and B3 stands for the coordinated strategy. The probabilities that the firm chooses these strategies are p(0 ≤ p ≤ 1), q(0 ≤ q ≤ 1), and 1-p-q.

The public’s strategy set is S_M_ = {M1, M2, M3}, where M1 represents the economic strategy, M2 represents the timeliness strategy, and M3 represents the coordinated strategy. The probabilities that the public chooses these strategies are u(0 ≤ u ≤ 1), v(0 ≤ v ≤ 1), and 1-u-v.

### Hypothesis 2

*Factors such as subsidies from the local government to enterprises and the public for the construction of major railroad projects affect the formation of and change in equilibrium* ^40^ *.*

The local government’s subsidy coefficient of α (0 ≤ α ≤ 1) to the enterprise is reflected in the subsidy or transaction share between the local government and the enterprise. When α = 0, the enterprise bears all the cost of emergency relief. When α = 1, the government bears all the cost of emergency relief, especially for the material supply enterprises. Hence, the subsidy coefficient can more intuitively describe the transaction share between both stakeholders.

The government’s subsidy coefficient β (0 ≤ β ≤ 1) to the public intuitively represents the transaction share between the local government and the public. β = 0 indicates that the enterprise bears all the emergency relief costs, and β = 1 indicates that the government bears all the emergency relief costs.

The three agents’ strategy choices are affected by reputational benefits, and θ1, θ2, and θ3 are the coefficients of the public good preferences of the local government, enterprises, and the public, respectively. The meanings of the other parameters and variables are shown in Table 1.

**Table 1:** Meaning of variables and parameters.

Parameter	Meaning	Value range
*C*_*11*_	The cost to the local government of choosing an economic strategy	*C*_*13*_ > *C*_*12*_ > *C*_*11*_ > *0*
*C*_*12*_	The cost to the local government of choosing a timeliness strategy	
*C*_*13*_	The cost to the local government of choosing a coordinated strategy	
*C*_*21*_	The cost to the enterprise of choosing an economic strategy	*C*_*23*_ > *C*_*22*_ > *C*_*21*_ > *0*
*C*_*22*_	The cost to the enterprise of choosing a timeliness strategy	
*C*_*23*_	The cost to the enterprise of choosing a coordinated strategy	
*α*	Government subsidy coefficient for enterprises	*0* ≤ *α* ≤ *1*
*C*_*31*_	The cost to the public of choosing an economic strategy	*C*_*32*_ > *C*_*33*_ > *C*_*31*_ > *0*
*C*_*32*_	The cost to the public of choosing a timeliness strategy	
*C*_*33*_	The cost to the public of choosing a coordinated strategy	
*β*	Government subsidy coefficient for public	*0* ≤ *β* ≤ *1*
*θ*_*1*_	Coefficient of local government interest preference	*0* ≤ *θ*_*1*_ ≤ *1*
*θ*_*2*_	Coefficient of enterprise interest preference	*0* ≤ *θ*_*2*_ ≤ *1*
*θ*_*3*_	Coefficient of public interest preference	*0* ≤ *θ*_*3*_ ≤ *1*
*R*_*11*_	Effect of economic strategy on local government reputation	*R*_*13*_ > *R*_*12*_ > *R*_*11*_ > *0*
*R*_*12*_	Effect of timeliness strategy on local government reputation	
*R*_*13*_	Effect of coordinated strategy on local government reputation	
*R*_*21*_	Effect of economic strategy on enterprise reputation	*R*_*23*_ > *R*_*22*_ > *R*_*21*_ > *0*
*R*_*22*_	Effect of timeliness strategy on enterprise reputation	
*R*_*23*_	Effect of coordinated strategy on enterprise reputation	
*R*_*31*_	Effect of economic strategy on public reputation	*R*_*32*_ > *R*_*33*_ > *R*_*31*_ > *0*
*R*_*32*_	Effect of timeliness strategy on public reputation	
*R*_*33*_	Effect of coordinated strategy on public reputation	

Value conditions such as *C*_*13*_ > *C*_*12*_ > *C*_*11*_ > *0* are set according to the mechanism design, extant literature^41^, and/or official data from General Office of the State Council of the People’s Republic of China.

## Model construction

The economic strategy focuses on cost minimization, resource optimization, and efficiency improvement. It emphasizes enhancing emergency response effectiveness through efficient resource management and cost control. The relevant parameters are *C11*, *C21*, *C31*, *R11*, *R21*, and *R31*. The timeliness strategy emphasizes the quick initiation and implementation of emergency responses, mainly by optimizing response time, resource deployment speed, and recovery time to improve efficiency. The relevant parameters are *C12*, *C22*, *C32*, *R12*, *R22*, and *R32*. The coordination strategy focuses on the collaboration of multiple agents, resource sharing, and information exchange. Its goal is to improve overall rescue effectiveness through cooperation and to promote the resource coordination and sharing of various parties. The relevant parameters are *C13*, *C23*, *C33*, *R13*, *R23*, and *R33*. Based on the model assumption above, the benefit matrix of the tripartite three-strategy collaborative decision-making model in this study is shown in Table 2.

**Table 2:** Returns of the three parties under each strategy.

		The public choosing an economic strategy	The public choosing a timeliness strategy	The public choosing a coordinated strategy
The local government choosing an economic strategy	The enterprise choosing an economic strategy	$$\left( {\begin{array}{*{20}c} { - C_{11} - \alpha C_{21} - \beta C_{31} + \theta_{1} R_{11} } \\ { - C_{21} + \alpha C_{21} + \theta_{2} R_{21} } \\ { - C_{31} + \beta C_{31} + \theta_{3} R_{31} } \\ \end{array} } \right)$$	$$\left( {\begin{array}{*{20}c} { - C_{11} - \alpha C_{21} - \beta C_{32} + \theta_{1} R_{11} } \\ { - C_{21} + \alpha C_{21} + \theta_{2} R_{21} } \\ { - C_{32} + \beta C_{32} + \theta_{3} R_{32} } \\ \end{array} } \right)$$	$$\left( {\begin{array}{*{20}c} { - C_{11} - \alpha C_{21} - \beta C_{33} + \theta_{1} R_{11} } \\ { - C_{21} + \alpha C_{21} + \theta_{2} R_{21} } \\ { - C_{33} + \beta C_{33} + \theta_{3} R_{33} } \\ \end{array} } \right)$$
	The enterprise choosing a timeliness strategy	$$\left( {\begin{array}{*{20}c} { - C_{11} - \alpha C_{{2{2}}} - \beta C_{31} + \theta_{1} R_{11} } \\ { - C_{22} + \alpha C_{22} + \theta_{2} R_{22} } \\ { - C_{31} + \beta C_{31} + \theta_{3} R_{31} } \\ \end{array} } \right)$$	$$\left( {\begin{array}{*{20}c} { - C_{11} - \alpha C_{22} - \beta C_{32} + \theta_{1} R_{11} } \\ { - C_{22} + \alpha C_{22} + \theta_{2} R_{22} } \\ { - C_{32} + \beta C_{32} + \theta_{3} R_{32} } \\ \end{array} } \right)$$	$$\left( {\begin{array}{*{20}c} { - C_{11} - \alpha C_{22} - \beta C_{33} + \theta_{1} R_{11} } \\ { - C_{22} + \alpha C_{22} + \theta_{2} R_{22} } \\ { - C_{33} + \beta C_{33} + \theta_{3} R_{33} } \\ \end{array} } \right)$$
	The enterprise choosing a coordinated strategy	$$\left( {\begin{array}{*{20}c} { - C_{11} - \alpha C_{23} - \beta C_{31} + \theta_{1} R_{11} } \\ { - C_{23} + \alpha C_{23} + \theta_{2} R_{23} } \\ { - C_{31} + \beta C_{31} + \theta_{3} R_{31} } \\ \end{array} } \right)$$	$$\left( {\begin{array}{*{20}c} { - C_{11} - \alpha C_{23} - \beta C_{32} + \theta_{1} R_{11} } \\ { - C_{23} + \alpha C_{23} + \theta_{2} R_{23} } \\ { - C_{32} + \beta C_{32} + \theta_{3} R_{32} } \\ \end{array} } \right)$$	$$\left( {\begin{array}{*{20}c} { - C_{11} - \alpha C_{23} - \beta C_{33} + \theta_{1} R_{11} } \\ { - C_{23} + \alpha C_{23} + \theta_{2} R_{23} } \\ { - C_{33} + \beta C_{33} + \theta_{3} R_{33} } \\ \end{array} } \right)$$
The enterprise choosing a timeliness strategy	The enterprise choosing an economic strategy	$$\left( {\begin{array}{*{20}c} { - C_{12} - \alpha C_{21} - \beta C_{31} + \theta_{1} R_{12} } \\ { - C_{21} + \alpha C_{21} + \theta_{2} R_{21} } \\ { - C_{31} + \beta C_{31} + \theta_{3} R_{31} } \\ \end{array} } \right)$$	$$\left( {\begin{array}{*{20}c} { - C_{12} - \alpha C_{21} - \beta C_{32} + \theta_{1} R_{12} } \\ { - C_{21} + \alpha C_{21} + \theta_{2} R_{21} } \\ { - C_{32} + \beta C_{32} + \theta_{3} R_{32} } \\ \end{array} } \right)$$	$$\left( {\begin{array}{*{20}c} { - C_{12} - \alpha C_{21} - \beta C_{33} + \theta_{1} R_{12} } \\ { - C_{21} + \alpha C_{21} + \theta_{2} R_{21} } \\ { - C_{33} + \beta C_{33} + \theta_{3} R_{33} } \\ \end{array} } \right)$$
	The enterprise choosing a timeliness strategy	$$\left( {\begin{array}{*{20}c} { - C_{12} - \alpha C_{22} - \beta C_{31} + \theta_{1} R_{12} } \\ { - C_{22} + \alpha C_{22} + \theta_{2} R_{22} } \\ { - C_{31} + \beta C_{31} + \theta_{3} R_{31} } \\ \end{array} } \right)$$	$$\left( {\begin{array}{*{20}c} { - C_{12} - \alpha C_{22} - \beta C_{32} + \theta_{1} R_{12} } \\ { - C_{22} + \alpha C_{22} + \theta_{2} R_{22} } \\ { - C_{32} + \beta C_{32} + \theta_{3} R_{32} } \\ \end{array} } \right)$$	$$\left( {\begin{array}{*{20}c} { - C_{12} - \alpha C_{22} - \beta C_{33} + \theta_{1} R_{12} } \\ { - C_{22} + \alpha C_{22} + \theta_{2} R_{22} } \\ { - C_{33} + \beta C_{33} + \theta_{3} R_{33} } \\ \end{array} } \right)$$
	The enterprise choosing a coordinated strategy	$$\left( {\begin{array}{*{20}c} { - C_{12} - \alpha C_{23} - \beta C_{31} + \theta_{1} R_{12} } \\ { - C_{23} + \alpha C_{23} + \theta_{2} R_{23} } \\ { - C_{31} + \beta C_{31} + \theta_{3} R_{31} } \\ \end{array} } \right)$$	$$\left( {\begin{array}{*{20}c} { - C_{12} - \alpha C_{23} - \beta C_{32} + \theta_{1} R_{12} } \\ { - C_{23} + \alpha C_{23} + \theta_{2} R_{23} } \\ { - C_{32} + \beta C_{32} + \theta_{3} R_{32} } \\ \end{array} } \right)$$	$$\left( {\begin{array}{*{20}c} { - C_{12} - \alpha C_{23} - \beta C_{{3{3}}} + \theta_{1} R_{12} } \\ { - C_{23} + \alpha C_{23} + \theta_{2} R_{23} } \\ { - C_{33} + \beta C_{33} + \theta_{3} R_{33} } \\ \end{array} } \right)$$
The enterprise choosing a coordinated strategy	The enterprise choosing an economic strategy	$$\left( {\begin{array}{*{20}c} { - C_{13} - \alpha C_{21} - \beta C_{31} + \theta_{1} R_{13} } \\ { - C_{21} + \alpha C_{21} + \theta_{2} R_{21} } \\ { - C_{31} + \beta C_{31} + \theta_{3} R_{31} } \\ \end{array} } \right)$$	$$\left( {\begin{array}{*{20}c} { - C_{13} - \alpha C_{21} - \beta C_{32} + \theta_{1} R_{13} } \\ { - C_{21} + \alpha C_{21} + \theta_{2} R_{21} } \\ { - C_{{3{2}}} + \beta C_{{3{2}}} + \theta_{3} R_{{3{2}}} } \\ \end{array} } \right)$$	$$\left( {\begin{array}{*{20}c} { - C_{13} - \alpha C_{21} - \beta C_{{3{3}}} + \theta_{1} R_{13} } \\ { - C_{21} + \alpha C_{21} + \theta_{2} R_{21} } \\ { - C_{{3{3}}} + \beta C_{{3{3}}} + \theta_{3} R_{{3{3}}} } \\ \end{array} } \right)$$
	The enterprise choosing a timeliness strategy	$$\left( {\begin{array}{*{20}c} { - C_{13} - \alpha C_{{2{2}}} - \beta C_{31} + \theta_{1} R_{13} } \\ { - C_{{2{2}}} + \alpha C_{{2{2}}} + \theta_{2} R_{{2{2}}} } \\ { - C_{31} + \beta C_{31} + \theta_{3} R_{31} } \\ \end{array} } \right)$$	$$\left( {\begin{array}{*{20}c} { - C_{13} - \alpha C_{{2{2}}} - \beta C_{{3{2}}} + \theta_{1} R_{13} } \\ { - C_{{2{2}}} + \alpha C_{{2{2}}} + \theta_{2} R_{{2{2}}} } \\ { - C_{{3{2}}} + \beta C_{{3{2}}} + \theta_{3} R_{{3{2}}} } \\ \end{array} } \right)$$	$$\left( {\begin{array}{*{20}c} { - C_{13} - \alpha C_{{2{2}}} - \beta C_{{3{3}}} + \theta_{1} R_{13} } \\ { - C_{{2{2}}} + \alpha C_{{2{2}}} + \theta_{2} R_{{2{2}}} } \\ { - C_{{3{3}}} + \beta C_{{3{3}}} + \theta_{3} R_{{3{3}}} } \\ \end{array} } \right)$$
	The enterprise choosing a coordinated strategy	$$\left( {\begin{array}{*{20}c} { - C_{13} - \alpha C_{{2{3}}} - \beta C_{31} + \theta_{1} R_{13} } \\ { - C_{{2{3}}} + \alpha C_{{2{3}}} + \theta_{2} R_{{2{3}}} } \\ { - C_{31} + \beta C_{31} + \theta_{3} R_{31} } \\ \end{array} } \right)$$	$$\left( {\begin{array}{*{20}c} { - C_{13} - \alpha C_{{2{3}}} - \beta C_{{3{2}}} + \theta_{1} R_{13} } \\ { - C_{{2{3}}} + \alpha C_{{2{3}}} + \theta_{2} R_{{2{3}}} } \\ { - C_{{3{2}}} + \beta C_{{3{2}}} + \theta_{3} R_{{3{2}}} } \\ \end{array} } \right)$$	$$\left( {\begin{array}{*{20}c} { - C_{13} - \alpha C_{{2{3}}} - \beta C_{{3{3}}} + \theta_{1} R_{13} } \\ { - C_{{2{3}}} + \alpha C_{{2{3}}} + \theta_{2} R_{{2{3}}} } \\ { - C_{{3{3}}} + \beta C_{{3{3}}} + \theta_{3} R_{{3{3}}} } \\ \end{array} } \right)$$

According to the strategy mix income in Table 2, the local government’s expectations in choosing the economic strategy, timeliness strategy, and coordinated strategy are

1 $$E_{x} = \left( {\theta_{1} R_{11} - \alpha C_{23} - \beta C_{33} - C_{11} } \right) + \alpha p\left( {C_{23} - C_{21} } \right) + \alpha p\left( {C_{23} - C_{22} } \right) + \beta u\left( {C_{33} - C_{31} } \right) + \beta v\left( {C_{33} - C_{32} } \right)$$

2 $$E_{y} = \left( {\theta_{1} R_{12} - \alpha C_{23} - \beta C_{33} - C_{12} } \right) + \alpha p\left( {C_{23} - C_{21} } \right) + \alpha p\left( {C_{23} - C_{22} } \right) + \beta u\left( {C_{33} - C_{31} } \right) + \beta v\left( {C_{33} - C_{32} } \right)$$

3 $$E_{1 - x - y} = \left( {\theta_{1} R_{13} - \alpha C_{23} - \beta C_{33} - C_{13} } \right) + \alpha p\left( {C_{23} - C_{21} } \right) + \alpha p\left( {C_{23} - C_{22} } \right) + \beta u\left( {C_{33} - C_{31} } \right) + \beta v\left( {C_{33} - C_{32} } \right)$$

The average expected revenue of a dependent territory government is

4 $$\begin{aligned} E_{A} & = x\left( {C_{13} - C_{11} + \theta_{1} C_{11} - \theta_{1} R_{13} } \right) + y\left( {C_{13} - C_{12} + \theta_{1} R_{12} - \theta_{1} R_{13} } \right) + \alpha p\left( {C_{23} - C_{21} } \right) \\ & \quad + \alpha q\left( {C_{23} - C_{22} } \right) + \beta u\left( {C_{33} - C_{31} } \right) + \beta v\left( {C_{33} - C_{32} } \right) + \left( {\theta_{1} R_{13} - \alpha C_{23} - \beta C_{33} - C_{13} } \right) \\ \end{aligned}$$

The expectations of enterprises in choosing economic, timeliness, and coordinated strategies are

5 $$E_{p} = \theta_{2} R_{21} - \alpha C_{21} - C_{21}$$

6 $$E_{q} = \theta_{2} R_{22} - \alpha C_{22} - C_{22}$$

7 $$E_{1 - p - q} = \theta_{2} R_{23} - \alpha C_{23} - C_{23}$$

The average expected income of enterprises is

8 $$E_{B} = p\left( {C_{23} - C_{21} + \alpha C_{23} - \alpha C_{21} + \theta_{2} R_{21} - \theta_{2} R_{23} } \right) + q\left( {C_{23} - C_{22} + \alpha C_{23} - \alpha C_{22} + \theta_{2} R_{22} - \theta_{2} R_{23} } \right) + \left( {\theta_{2} R_{23} - \alpha C_{23} - C_{23} } \right)$$

The public’s expectations in choosing the economic strategy, timeliness strategy, and coordinated strategy are

9 $$E_{u} = \beta C_{31} - C_{31} - \theta_{3} R_{31}$$

10 $$E_{v} = \beta C_{32} - C_{32} - \theta_{3} R_{32}$$

11 $$E_{1 - u - v} = \beta C_{33} - C_{33} - \theta_{3} R_{33}$$

The public’s average expected benefit is

12 $$E_{M} = u\left( {\left( {1 - \beta } \right)\left( {C_{33} - C_{31} } \right) + \theta_{3} (R_{31} - R_{33} )} \right) + v\left( {\left( {1 - \beta } \right)\left( {C_{33} - C_{32} } \right) + \theta_{3} (R_{32} - R_{33} )} \right) + \left( {\theta_{3} R_{33} + \beta C_{33} - C_{33} } \right)$$

A replicator dynamic equation is used to simulate the long-term evolution of strategies in a multi-agent environment. In EGT, the replicator dynamic model describes how participants adjust the probability of selecting a strategy based on the past success or failure of that strategy^16^. In emergency response, each agent’s strategy choice is influenced by its past performance: strategies with good performance (i.e., efficient responses) increase their probability of being selected, while underperforming strategies are gradually eliminated.

The replicator dynamic equation for the local government, enterprises, and the public is given as:

13 $$\frac{dr}{{dt}}{\text{ = r}} \cdot {(1} - {\text{r)}} \cdot {\text{(E}}_{{\text{r}}} - {\text{E}}_{{{\text{avg}}}} )$$

where r represents the probability of selecting a certain strategy, E_r_ is strategy r’s payoff, and E_avg_ is the average payoff of all strategies.

This equation describes how each participant adapts its strategy over time in response to environmental changes, such as subsidy policies and societal demands. Based on the above results, the local government, enterprises, and the public can copy the dynamic equation, and the tripartite three-strategy collaborative decision-making system can be obtained as

$$\left\{\begin{array}{c}{f}_{1}\left(x\right)=\frac{dx}{dt}=x\left(\left(1-x\right)\left({C}_{13}-{C}_{11}+{\theta }_{1}{R}_{11}-{\theta }_{1}{R}_{13}\right)-y\left({C}_{13}-{C}_{12}+{\theta }_{1}{R}_{12}-{\theta }_{1}{R}_{13}\right)\right) \\ {f}_{2}\left(y\right)=\frac{dy}{dt}=y\left(\left(1-y\right)\left({C}_{13}-{C}_{11}+{\theta }_{1}{R}_{12}-{\theta }_{1}{R}_{13}\right)-y\left({C}_{13}-{C}_{11}+{\theta }_{1}{R}_{11}-{\theta }_{1}{R}_{13}\right)\right) \\ {f}_{3}\left(p\right)=\frac{dp}{dt}=p\left(\left(1-p\right)\left({C}_{23}-{C}_{21}+\alpha {C}_{23}-\alpha {C}_{21}+{\theta }_{2}{R}_{21}-{\theta }_{2}{R}_{23}\right)-q\left({C}_{23}-{C}_{21}+\alpha {C}_{23}-\alpha {C}_{21}+{\theta }_{2}{R}_{22}-{\theta }_{2}{R}_{23}\right)\right)\\ {f}_{4}\left(q\right)=\frac{dq}{dt}=q\left(\left(1-q\right)\left({C}_{23}-{C}_{22}+\alpha {C}_{23}-\alpha {C}_{22}+{\theta }_{2}{R}_{22}-{\theta }_{2}{R}_{23}\right)-p\left({C}_{23}-{C}_{21}+\alpha {C}_{23}-\alpha {C}_{21}+{\theta }_{2}{R}_{21}-{\theta }_{2}{R}_{23}\right)\right)\\ {f}_{5}\left(u\right)=\frac{du}{dt}=u\left(\left(1-u\right)\left({C}_{33}-{C}_{31}+{\beta {C}_{31}-\beta {C}_{33}+\theta }_{3}{R}_{31}-{\theta }_{3}{R}_{33}\right)-v\left({C}_{33}-{C}_{32}+{\beta {C}_{32}-\beta {C}_{33}+\theta }_{3}{R}_{32}-{\theta }_{3}{R}_{33}\right)\right)\\ {f}_{6}\left(v\right)=\frac{dv}{dt}=v\left(\left(1-v\right)\left({C}_{33}-{C}_{32}+{\beta {C}_{32}-\beta {C}_{33}+\theta }_{3}{R}_{32}-{\theta }_{3}{R}_{33}\right)-u\left({C}_{33}-{C}_{31}+{\beta {C}_{31}-\beta {C}_{33}+\theta }_{3}{R}_{31}-{\theta }_{3}{R}_{33}\right)\right)\end{array}\right.$$

Based on the above multivariate partial differential equations, the Jacobian matrix J of the asymmetric evolutionary game model for cooperative decision-making of localized emergency response in railroad engineering construction can be further obtained, where df_1_(x)/dx represents the derivative of f_1_(x) with respect to x and ∂f_2_(y)/∂x represents the partial derivative of f_2_(y) with respect to x.

Since x and y, p and q, or u and v cannot be 1 simultaneously, 27 equilibrium points exist in the asymmetric evolutionary game model of cooperative decision-making for localized emergency response in railroad engineering construction, which are calculated as E1 ~ E27. The corresponding eigenvalues can be obtained by substituting the equilibrium points into the Jacobian matrix, and the eigenvalues represent the agent’s relative profits in the selection of strategies. The long-term evolution characteristics of the agent evolutionary game system at the internal equilibrium point of each group of pure strategies depend on these six groups of eigenvalue parameters, as shown in Table 3.

**Table 3:** Eigenvalue parameters of the tripartite three-strategy cooperative decision system.

Equilibrium point	Equilibrium point coordinates	The eigenvalue of the corresponding matrix after each equilibrium point is substituted		
		λ_1_	λ_2_	λ_3_
1	(0,0,0,0,0,0)	$C_{13} - C_{11} + \theta_{1} R_{11} - \theta_{1} R_{13}$	$C_{13} - C_{12} + \theta_{1} R_{12} - \theta_{1} R_{13}$	$C_{23} - C_{21} - \alpha C_{21} + \alpha C_{23}$ $+ \theta_{2} R_{21} - \theta_{2} R_{23}$
2	(1,0,0,0,0,0)	$C_{11} - C_{12} - \theta_{1} R_{11} + \theta_{1} R_{12}$	$C_{11} - C_{13} - \theta_{1} R_{11} + \theta_{1} R_{13}$	$C_{23} - C_{21} - \alpha C_{21} + \alpha C_{23}$ $+ \theta_{2} R_{21} - \theta_{2} R_{23}$
3	(0,1,0,0,0,0)	$C_{12} - C_{11} + \theta_{1} R_{11} - \theta_{1} R_{12}$	$C_{12} - C_{13} - \theta_{1} R_{12} + \theta_{1} R_{13}$	$C_{23} - C_{21} - \alpha C_{21} + \alpha C_{23}$ $+ \theta_{2} R_{21} - \theta_{2} R_{23}$
4	(0,0,1,0,0,0)	$C_{13} - C_{11} + \theta_{1} R_{11} - \theta_{1} R_{13}$	$C_{13} - C_{12} + \theta_{1} R_{12} - \theta_{1} R_{13}$	$C_{21} - C_{22} + \alpha C_{21} - \alpha C_{22}$ $- \theta_{2} R_{21} + \theta_{2} R_{22}$
5	(0,0,0,1,0,0)	$C_{13} - C_{11} + \theta_{1} R_{11} - \theta_{1} R_{13}$	$C_{13} - C_{12} + \theta_{1} R_{12} - \theta_{1} R_{13}$	$C_{22} - C_{21} - \alpha C_{21} + \alpha C_{22}$ $+ \theta_{2} R_{21} - \theta_{2} R_{22}$
6	(0,0,0,0,1,0)	$C_{13} - C_{11} + \theta_{1} R_{11} - \theta_{1} R_{13}$	$C_{13} - C_{12} + \theta_{1} R_{12} - \theta_{1} R_{13}$	$C_{23} - C_{21} - \alpha C_{21} + \alpha C_{23}$ $+ \theta_{2} R_{21} - \theta_{2} R_{23}$
7	(1,0,1,0,0,0)	$C_{11} - C_{12} - \theta_{1} R_{11} + \theta_{1} R_{12}$	$C_{11} - C_{13} - \theta_{1} R_{11} + \theta_{1} R_{13}$	$C_{21} - C_{22} + \alpha C_{21} - \alpha C_{22}$ $- \theta_{2} R_{21} + \theta_{2} R_{22}$
8	(0,0,0,0,0,1)	$C_{13} - C_{11} + \theta_{1} R_{11} - \theta_{1} R_{13}$	$C_{13} - C_{12} + \theta_{1} R_{12} - \theta_{1} R_{13}$	$C_{23} - C_{21} - \alpha C_{21} + \alpha C_{23}$ $+ \theta_{2} R_{21} - \theta_{2} R_{23}$
9	(0,1,1,0,0,0)	$C_{12} - C_{11} + \theta_{1} R_{11} - \theta_{1} R_{12}$	$C_{12} - C_{13} - \theta_{1} R_{12} + \theta_{1} R_{13}$	$C_{21} - C_{22} + \alpha C_{21} - \alpha C_{22}$ $- \theta_{2} R_{21} + \theta_{2} R_{22}$
10	(1,0,0,1,0,0)	$C_{11} - C_{12} - \theta_{1} R_{11} + \theta_{1} R_{12}$	$C_{11} - C_{13} - \theta_{1} R_{11} + \theta_{1} R_{13}$	$C_{22} - C_{21} - \alpha C_{21} + \alpha C_{22}$ $+ \theta_{2} R_{21} - \theta_{2} R_{22}$
11	(0,1,0,1,0,0)	$C_{12} - C_{11} + \theta_{1} R_{11} - \theta_{1} R_{12}$	$C_{12} - C_{13} - \theta_{1} R_{12} + \theta_{1} R_{13}$	$C_{22} - C_{21} - \alpha C_{21} + \alpha C_{22}$ $+ \theta_{2} R_{21} - \theta_{2} R_{22}$
12	(1,0,0,0,1,0)	$C_{11} - C_{12} - \theta_{1} R_{11} + \theta_{1} R_{12}$	$C_{11} - C_{13} - \theta_{1} R_{11} + \theta_{1} R_{13}$	$C_{23} - C_{21} - \alpha C_{21} + \alpha C_{23}$ $+ \theta_{2} R_{21} - \theta_{2} R_{23}$
13	(0,1,0,0,1,0)	$C_{12} - C_{11} + \theta_{1} R_{11} - \theta_{1} R_{12}$	$C_{12} - C_{13} - \theta_{1} R_{12} + \theta_{1} R_{13}$	$C_{23} - C_{21} - \alpha C_{21} + \alpha C_{23}$ $+ \theta_{2} R_{21} - \theta_{2} R_{23}$
14	(1,0,0,0,0,1)	$C_{11} - C_{12} - \theta_{1} R_{11} + \theta_{1} R_{12}$	$C_{11} - C_{13} - \theta_{1} R_{11} + \theta_{1} R_{13}$	$C_{23} - C_{21} - \alpha C_{21} + \alpha C_{23}$ $+ \theta_{2} R_{21} - \theta_{2} R_{23}$
15	(0,0,1,0,1,0)	$C_{12} - C_{11} + \theta_{1} R_{11} - \theta_{1} R_{12}$	$C_{13} - C_{12} + \theta_{1} R_{12} - \theta_{1} R_{13}$	$C_{21} - C_{22} + \alpha C_{21} - \alpha C_{22}$ $- \theta_{2} R_{21} + \theta_{2} R_{22}$
16	(0,1,0,0,0,1)	$C_{12} - C_{11} + \theta_{1} R_{11} - \theta_{1} R_{12}$	$C_{12} - C_{13} - \theta_{1} R_{12} + \theta_{1} R_{13}$	$C_{23} - C_{21} - \alpha C_{21} + \alpha C_{23}$ $+ \theta_{2} R_{21} - \theta_{2} R_{23}$
17	(0,0,0,1,1,0)	$C_{12} - C_{11} + \theta_{1} R_{11} - \theta_{1} R_{12}$	$C_{13} - C_{12} + \theta_{1} R_{12} - \theta_{1} R_{13}$	$C_{22} - C_{21} - \alpha C_{21} + \alpha C_{22}$ $+ \theta_{2} R_{21} - \theta_{2} R_{22}$
18	(0,0,1,0,0,1)	$C_{12} - C_{11} + \theta_{1} R_{11} - \theta_{1} R_{12}$	$C_{13} - C_{12} + \theta_{1} R_{12} - \theta_{1} R_{13}$	$C_{21} - C_{22} + \alpha C_{21} - \alpha C_{22}$ $- \theta_{2} R_{21} + \theta_{2} R_{22}$
19	(0,0,0,1,0,1)	$C_{12} - C_{11} + \theta_{1} R_{11} - \theta_{1} R_{12}$	$C_{13} - C_{12} + \theta_{1} R_{12} - \theta_{1} R_{13}$	$C_{22} - C_{21} - \alpha C_{21} + \alpha C_{22}$ $+ \theta_{2} R_{21} - \theta_{2} R_{22}$
20	(1,0,1,0,1,0)	$C_{11} - C_{12} - \theta_{1} R_{11} + \theta_{1} R_{12}$	$C_{11} - C_{13} - \theta_{1} R_{11} + \theta_{1} R_{13}$	$C_{21} - C_{22} + \alpha C_{21} - \alpha C_{22}$ $- \theta_{2} R_{21} + \theta_{2} R_{22}$
21	(0,1,1,0,1,0)	$C_{12} - C_{11} + \theta_{1} R_{11} - \theta_{1} R_{12}$	$C_{12} - C_{13} R_{12} + \theta_{1} R_{13}$	$C_{21} - C_{22} + \alpha C_{21} - \alpha C_{22}$ $- \theta_{2} R_{21} + \theta_{2} R_{22}$
22	(1,0,0,1,1,0)	$C_{11} - C_{12} - \theta_{1} R_{11} + \theta_{1} R_{12}$	$C_{11} - C_{13} - \theta_{1} R_{11} + \theta_{1} R_{13}$	$C_{22} - C_{21} - \alpha C_{21} + \alpha C_{22}$ $+ \theta_{2} R_{21} - \theta_{2} R_{22}$
23	(1,0,1,0,0,1)	$C_{11} - C_{12} - \theta_{1} R_{11} + \theta_{1} R_{12}$	$C_{11} - C_{13} - \theta_{1} R_{11} + \theta_{1} R_{13}$	$C_{21} - C_{22} + \alpha C_{21} - \alpha C_{22}$ $- \theta_{2} R_{21} + \theta_{2} R_{22}$
24	(0,1,0,1,1,0)	$C_{12} - C_{11} + \theta_{1} R_{11} - \theta_{1} R_{12}$	$C_{12} - C_{13} - \theta_{1} R_{12} + \theta_{1} R_{13}$	$C_{22} - C_{21} - \alpha C_{21} + \alpha C_{22}$ $+ \theta_{2} R_{21} - \theta_{2} R_{22}$
25	(0,1,1,0,0,1)	$C_{12} - C_{11} + \theta_{1} R_{11} - \theta_{1} R_{12}$	$C_{12} - C_{13} - \theta_{1} R_{12} + \theta_{1} R_{13}$	$C_{21} - C_{22} + \alpha C_{21} - \alpha C_{22}$ $- \theta_{2} R_{21} + \theta_{2} R_{22}$
26	(1,0,0,1,0,1)	$C_{11} - C_{12} - \theta_{1} R_{11} + \theta_{1} R_{12}$	$C_{11} - C_{13} - \theta_{1} R_{11} + \theta_{1} R_{13}$	$C_{22} - C_{21} - \alpha C_{21} + \alpha C_{22}$ $+ \theta_{2} R_{21} - \theta_{2} R_{22}$
27	(0,1,0,1,0,1)	$C_{12} - C_{11} + \theta_{1} R_{11} - \theta_{1} R_{12}$	$C_{12} - C_{13} - \theta_{1} R_{12} + \theta_{1} R_{13}$	$C_{22} - C_{21} - \alpha C_{21} + \alpha C_{22}$ $+ \theta_{2} R_{21} - \theta_{2} R_{22}$

Equilibrium point	Equilibrium point coordinates	The eigenvalue of the corresponding matrix after each equilibrium point is substituted		
		λ_4_	λ_5_	λ_6_
1	(0,0,0,0,0,0)	$C_{23} - C_{22} - \alpha C_{22} + \alpha C_{23}$ $+ \theta_{2} R_{22} - \theta_{2} R_{23}$	$C_{33} - C_{31} + \beta C_{31} - \beta C_{33}$ $+ \theta_{3} R_{31} - \theta_{3} R_{33}$	$C_{33} - C_{32} + \beta C_{32} - \beta C_{33}$ $+ \theta_{3} R_{32} - \theta_{3} R_{33}$
2	(1,0,0,0,0,0)	$C_{23} - C_{22} - \alpha C_{22} + \alpha C_{23}$ $+ \theta_{2} R_{22} - \theta_{2} R_{23}$	$C_{33} - C_{31} + \beta C_{31} - \beta C_{33}$ $+ \theta_{3} R_{31} - \theta_{3} R_{33}$	$C_{33} - C_{32} + \beta C_{32} - \beta C_{33}$ $+ \theta_{3} R_{32} - \theta_{3} R_{33}$
3	(0,1,0,0,0,0)	$C_{23} - C_{22} - \alpha C_{22} + \alpha C_{23}$ $+ \theta_{2} R_{22} - \theta_{2} R_{23}$	$C_{33} - C_{31} + \beta C_{31} - \beta C_{33}$ $+ \theta_{3} R_{31} - \theta_{3} R_{33}$	$C_{33} - C_{32} + \beta C_{32} - \beta C_{33}$ $+ \theta_{3} R_{32} - \theta_{3} R_{33}$
4	(0,0,1,0,0,0)	$C_{21} - C_{23} + \alpha C_{21} - \alpha C_{23}$ $- \theta_{2} R_{21} + \theta_{2} R_{23}$	$C_{33} - C_{31} + \beta C_{31} - \beta C_{33}$ $+ \theta_{3} R_{31} - \theta_{3} R_{33}$	$C_{33} - C_{32} + \beta C_{32} - \beta C_{33}$ $+ \theta_{3} R_{32} - \theta_{3} R_{33}$
5	(0,0,0,1,0,0)	$C_{22} - C_{23} + \alpha C_{22} - \alpha C_{23}$ $- \theta_{2} R_{22} + \theta_{2} R_{23}$	$C_{33} - C_{31} + \beta C_{31} - \beta C_{33}$ $+ \theta_{3} R_{31} - \theta_{3} R_{33}$	$C_{33} - C_{32} + \beta C_{32} - \beta C_{33}$ $+ \theta_{3} R_{32} - \theta_{3} R_{33}$
6	(0,0,0,0,1,0)	$C_{23} - C_{22} - \alpha C_{22} + \alpha C_{23}$ $+ \theta_{2} R_{22} - \theta_{2} R_{23}$	$C_{31} - C_{32} - \beta C_{31} + \beta C_{32}$ $- \theta_{3} R_{31} + \theta_{3} R_{32}$	$C_{31} - C_{33} - \beta C_{31} + \beta C_{33}$ $- \theta_{3} R_{31} + \theta_{3} R_{33}$
7	(1,0,1,0,0,0)	$C_{21} - C_{23} + \alpha C_{21} - \alpha C_{23}$ $- \theta_{2} R_{21} + \theta_{2} R_{23}$	$C_{33} - C_{31} + \beta C_{31} - \beta C_{33}$ $+ \theta_{3} R_{31} - \theta_{3} R_{33}$	$C_{33} - C_{32} + \beta C_{32} - \beta C_{33}$ $+ \theta_{3} R_{32} - \theta_{3} R_{33}$
8	(0,0,0,0,0,1)	$C_{23} - C_{22} - \alpha C_{22} + \alpha C_{23}$ $+ \theta_{2} R_{22} - \theta_{2} R_{23}$	$C_{32} - C_{31} + \beta C_{31} - \beta C_{32}$ $+ \theta_{3} R_{31} - \theta_{3} R_{32}$	$C_{32} - C_{33} - \beta C_{32} + \beta C_{33}$ $- \theta_{3} R_{32} + \theta_{3} R_{33}$
9	(0,1,1,0,0,0)	$C_{21} - C_{23} + \alpha C_{21} - \alpha C_{23}$ $- \theta_{2} R_{21} + \theta_{2} R_{23}$	$C_{33} - C_{31} + \beta C_{31} - \beta C_{33}$ $+ \theta_{3} R_{31} - \theta_{3} R_{33}$	$C_{33} - C_{32} + \beta C_{32} - \beta C_{33}$ $+ \theta_{3} R_{32} - \theta_{3} R_{33}$
10	(1,0,0,1,0,0)	$C_{22} - C_{23} + \alpha C_{22} - \alpha C_{23}$ $- \theta_{2} R_{22} + \theta_{2} R_{23}$	$C_{33} - C_{31} + \beta C_{31} - \beta C_{33}$ $+ \theta_{3} R_{31} - \theta_{3} R_{33}$	$C_{33} - C_{32} + \beta C_{32} - \beta C_{33}$ $+ \theta_{3} R_{32} - \theta_{3} R_{33}$
11	(0,1,0,1,0,0)	$C_{22} - C_{23} + \alpha C_{22} - \alpha C_{23}$ $- \theta_{2} R_{22} + \theta_{2} R_{23}$	$C_{33} - C_{31} + \beta C_{31} - \beta C_{33}$ $+ \theta_{3} R_{31} - \theta_{3} R_{33}$	$C_{33} - C_{32} + \beta C_{32} - \beta C_{33}$ $+ \theta_{3} R_{32} - \theta_{3} R_{33}$
12	(1,0,0,0,1,0)	$C_{23} - C_{22} - \alpha C_{22} + \alpha C_{23}$ $+ \theta_{2} R_{22} - \theta_{2} R_{23}$	$C_{31} - C_{32} - \beta C_{31} + \beta C_{32}$ $- \theta_{3} R_{31} + \theta_{3} R_{32}$	$C_{31} - C_{33} - \beta C_{31} + \beta C_{33}$ $- \theta_{3} R_{31} + \theta_{3} R_{33}$
13	(0,1,0,0,1,0)	$C_{23} - C_{22} - \alpha C_{22} + \alpha C_{23}$ $+ \theta_{2} R_{22} - \theta_{2} R_{23}$	$C_{31} - C_{32} - \beta C_{31} + \beta C_{32}$ $- \theta_{3} R_{31} + \theta_{3} R_{32}$	$C_{31} - C_{33} - \beta C_{31} + \beta C_{33}$ $- \theta_{3} R_{31} + \theta_{3} R_{33}$
14	(1,0,0,0,0,1)	$C_{23} - C_{22} - \alpha C_{22} + \alpha C_{23}$ $+ \theta_{2} R_{22} - \theta_{2} R_{23}$	$C_{32} - C_{31} + \beta C_{31} - \beta C_{32}$ $+ \theta_{3} R_{31} - \theta_{3} R_{32}$	$C_{32} - C_{33} - \beta C_{32} + \beta C_{33}$ $- \theta_{3} R_{32} + \theta_{3} R_{33}$
15	(0,0,1,0,1,0)	$C_{21} - C_{23} + \alpha C_{21} - \alpha C_{23}$ $- \theta_{2} R_{21} + \theta_{2} R_{23}$	$C_{31} - C_{32} - \beta C_{31} + \beta C_{32}$ $- \theta_{3} R_{31} + \theta_{3} R_{32}$	$C_{31} - C_{33} - \beta C_{31} + \beta C_{33}$ $- \theta_{3} R_{31} + \theta_{3} R_{33}$
16	(0,1,0,0,0,1)	$C_{23} - C_{22} - \alpha C_{22} + \alpha C_{23}$ $+ \theta_{2} R_{22} - \theta_{2} R_{23}$	$C_{32} - C_{31} + \beta C_{31} - \beta C_{32}$ $+ \theta_{3} R_{31} - \theta_{3} R_{32}$	$C_{32} - C_{33} - \beta C_{32} + \beta C_{33}$ $- \theta_{3} R_{32} + \theta_{3} R_{33}$
17	(0,0,0,1,1,0)	$C_{22} - C_{23} + \alpha C_{22} - \alpha C_{23}$ $- \theta_{2} R_{22} + \theta_{2} R_{23}$	$C_{31} - C_{32} - \beta C_{31} + \beta C_{32}$ $- \theta_{3} R_{31} + \theta_{3} R_{32}$	$C_{31} - C_{33} - \beta C_{31} + \beta C_{33}$ $- \theta_{3} R_{31} + \theta_{3} R_{33}$
18	(0,0,1,0,0,1)	$C_{21} - C_{23} + \alpha C_{21} - \alpha C_{23}$ $- \theta_{2} R_{21} + \theta_{2} R_{23}$	$C_{32} - C_{31} + \beta C_{31} - \beta C_{32}$ $+ \theta_{3} R_{31} - \theta_{3} R_{32}$	$C_{32} - C_{33} - \beta C_{32} + \beta C_{33}$ $- \theta_{3} R_{32} + \theta_{3} R_{33}$
19	(0,0,0,1,0,1)	$C_{22} - C_{23} + \alpha C_{22} - \alpha C_{23}$ $- \theta_{2} R_{22} + \theta_{2} R_{23}$	$C_{32} - C_{31} + \beta C_{31} - \beta C_{32}$ $+ \theta_{3} R_{31} - \theta_{3} R_{32}$	$C_{32} - C_{33} - \beta C_{32} + \beta C_{33}$ $- \theta_{3} R_{32} + \theta_{3} R_{33}$
20	(1,0,1,0,1,0)	$C_{21} - C_{23} + \alpha C_{21} - \alpha C_{23}$ $- \theta_{2} R_{21} + \theta_{2} R_{23}$	$C_{31} - C_{32} - \beta C_{31} + \beta C_{32}$ $- \theta_{3} R_{31} + \theta_{3} R_{32}$	$C_{31} - C_{33} - \beta C_{31} + \beta C_{33}$ $- \theta_{3} R_{31} + \theta_{3} R_{33}$
21	(0,1,1,0,1,0)	$C_{21} - C_{23} + \alpha C_{21} - \alpha C_{23}$ $- \theta_{2} R_{21} + \theta_{2} R_{23}$	$C_{31} - C_{32} - \beta C_{31} + \beta C_{32}$ $- \theta_{3} R_{31} + \theta_{3} R_{32}$	$C_{31} - C_{33} - \beta C_{31} + \beta C_{33}$ $- \theta_{3} R_{31} + \theta_{3} R_{33}$
22	(1,0,0,1,1,0)	$C_{22} - C_{23} + \alpha C_{22} - \alpha C_{23}$ $- \theta_{2} R_{22} + \theta_{2} R_{23}$	$C_{31} - C_{32} - \beta C_{31} + \beta C_{32}$ $- \theta_{3} R_{31} + \theta_{3} R_{32}$	$C_{31} - C_{33} - \beta C_{31} + \beta C_{33}$ $- \theta_{3} R_{31} + \theta_{3} R_{33}$
23	(1,0,1,0,0,1)	$C_{21} - C_{23} + \alpha C_{21} - \alpha C_{23}$ $- \theta_{2} R_{21} + \theta_{2} R_{23}$	$C_{32} - C_{31} + \beta C_{31} - \beta C_{32}$ $+ \theta_{3} R_{31} - \theta_{3} R_{32}$	$C_{32} - C_{33} - \beta C_{32} + \beta C_{33}$ $- \theta_{3} R_{32} + \theta_{3} R_{33}$
24	(0,1,0,1,1,0)	$C_{22} - C_{23} + \alpha C_{22} - \alpha C_{23}$ $- \theta_{2} R_{22} + \theta_{2} R_{23}$	$C_{31} - C_{32} - \beta C_{31} + \beta C_{32}$ $- \theta_{3} R_{31} + \theta_{3} R_{32}$	$C_{31} - C_{33} - \beta C_{31} + \beta C_{33}$ $- \theta_{3} R_{31} + \theta_{3} R_{33}$
25	(0,1,1,0,0,1)	$C_{21} - C_{23} + \alpha C_{21} - \alpha C_{23}$ $- \theta_{2} R_{21} + \theta_{2} R_{23}$	$C_{32} - C_{31} + \beta C_{31} - \beta C_{32}$ $+ \theta_{3} R_{31} - \theta_{3} R_{32}$	$C_{32} - C_{33} - \beta C_{32} + \beta C_{33}$ $- \theta_{3} R_{32} + \theta_{3} R_{33}$
26	(1,0,0,1,0,1)	$C_{22} - C_{23} + \alpha C_{22} - \alpha C_{23}$ $- \theta_{2} R_{22} + \theta_{2} R_{23}$	$C_{32} - C_{31} + \beta C_{31} - \beta C_{32}$ $+ \theta_{3} R_{31} - \theta_{3} R_{32}$	$C_{32} - C_{33} - \beta C_{32} + \beta C_{33}$ $- \theta_{3} R_{32} + \theta_{3} R_{33}$
27	(0,1,0,1,0,1)	$C_{22} - C_{23} + \alpha C_{22} - \alpha C_{23}$ $- \theta_{2} R_{22} + \theta_{2} R_{23}$	$C_{32} - C_{31} + \beta C_{31} - \beta C_{32}$ $+ \theta_{3} R_{31} - \theta_{3} R_{32}$	$C_{32} - C_{33} - \beta C_{32} + \beta C_{33}$ $- \theta_{3} R_{32} + \theta_{3} R_{33}$

$$J=\left[\begin{array}{c}\frac{d{f}_{1}(x)}{dx} \frac{\partial {f}_{1}(x)}{\partial y} \frac{\partial {f}_{1}(x)}{\partial p} \frac{\partial {f}_{1}(x)}{\partial q} \frac{\partial {f}_{1}(x)}{\partial u} \frac{\partial {f}_{1}(x)}{\partial v}\\ \frac{\partial {f}_{2}(y)}{\partial x} \frac{d{f}_{2}(y)}{dy} \frac{\partial {f}_{2}(y)}{\partial p} \frac{\partial {f}_{2}(y)}{\partial q} \frac{\partial {f}_{2}(y)}{\partial u} \frac{\partial {f}_{2}(y)}{\partial v}\\ \frac{\partial {f}_{3}(p)}{\partial x} \frac{\partial {f}_{3}(p)}{\partial y} \frac{d{f}_{3}(p)}{dp} \frac{\partial {f}_{3}(p)}{\partial q} \frac{\partial {f}_{3}(p)}{\partial u} \frac{\partial {f}_{3}(p)}{\partial v}\\ \frac{\partial {f}_{4}(q)}{\partial x} \frac{\partial {f}_{4}(q)}{\partial y} \frac{\partial {f}_{4}(q)}{\partial p} \frac{d{f}_{4}(q)}{dq} \frac{\partial {f}_{4}(q)}{\partial u} \frac{\partial {f}_{4}(q)}{\partial v}\\ \frac{\partial {f}_{5}(u)}{\partial x} \frac{\partial {f}_{5}(u)}{\partial y} \frac{\partial {f}_{5}(u)}{\partial p} \frac{\partial {f}_{5}(u)}{\partial q} \frac{d{f}_{5}(u)}{du} \frac{\partial {f}_{5}(u)}{\partial v}\\ \frac{\partial {f}_{6}(v)}{\partial x} \frac{\partial {f}_{6}(v)}{\partial y} \frac{\partial {f}_{6}(v)}{\partial p} \frac{\partial {f}_{6}(v)}{\partial q} \frac{\partial {f}_{6}(v)}{\partial u} \frac{d{f}_{6}(v)}{dv}\end{array}\right]$$

### Evolutionary stability strategy analysis

Through the tripartite three-strategy cooperative decision-making system, when f_1_(x) = f_2_(y) = f_3_(p) = f_4_(q) = f_5_(u) = f_6_(v) = 0, the change rate of system strategy selection is 0. According to Table 3, there are a total of nine groups of non-repeated eigenvalue parameters in the tripartite and three-strategy collaborative decision-making system for localized emergency response of major railroad projects (eigenvalue parameters with the same absolute value are defined as repeated eigenvalue parameters). That is, the main body selection selects nine groups of relative profit parameters, and six groups of relative profit parameters determine the system’s long-term evolution characteristics at the equilibrium point. Therefore, the long-term stable evolution equilibrium in the system contains 2^9^ complicated game scenarios.

Based on the eigenvalues of the equilibrium point corresponding to the Jacobian matrix in Table 3, the equilibrium point’s state is analyzed according to the scenario selected by the strategy. The combination of strategies discusses the stability of equilibrium points in a tripartite cooperative decision-making system.

#### Scenario 1

Local governments, enterprises, and the public jointly choose economic strategies for constructing major railroad projects. The parameters are adjusted to achieve long-term evolutionary stability of the multi-agent co-selected economic strategy combination so that the pure strategy’s internal equilibrium point E_20_(1,0,1,0,1,0) is the evolutionarily stable equilibrium point. When the local government chooses the economic strategy, the conditions (C11-C12) < θ1(R11-R12) and (C11-C13) < θ1(R11-R13) are met; that is, the cost difference between the economic strategy and the timeliness strategy is smaller than the local government’s reputation income difference. The cost difference between the economic and coordinated strategies is smaller than the difference in the local government’s reputation income.

Moreover, when an enterprise chooses an economic strategy, (C21-C22)(1 + α) < θ2(R21-R22) and (C21-C23)(1 + α) < θ2(R21-R23); that is, the sum of the cost difference between the economic strategy and the timeliness strategy of the enterprise and the government subsidies obtained by the enterprise in choosing the economic strategy. Moreover, the sum of the cost difference between the economic strategy and the coordinated strategy and the government subsidies obtained by the economic strategy is less than the reputation income difference under the corporate strategy choice.

Moreover, the public also chooses an economic strategy. At this time, the pure strategy’s internal equilibrium point must meet the conditions (C31-C32)(1-β) < θ3(R31-R32) and (C31-C33)(1-β) < θ3(R31-R33), and the eigenvalues of the Jacobian matrix corresponding to the equilibrium point E_20_ meet the condition that all of them are less than 0. At this point, the system achieves long-term stable equilibrium (ESE).

#### Scenario 2

The local government, enterprises, and the public jointly choose the timeliness strategy for constructing major railroad projects. To achieve long-term evolutionary stability of the strategy combination, the pure strategy’s internal equilibrium point E_27_ (0,1,0,1,0,1) is the evolution-stable equilibrium point. In this case, the Jacobian matrix’s eigenvalues corresponding to the equilibrium point E_27_ must meet the condition that all are less than 0 so that the system obtains long-term ESE at this point. The three strategies for a collaborative decision-making system satisfy the following conditions: (C12-C11) < θ1(R12-R11), (C12-C13) < θ1(R12-R13), (C22-C21)(1 + α) < θ2(R22-R21), (C22-C23)(1 + α) < θ2(R22-R23), (C32-C31)(1-β) < θ3(R32-R31), and (C32-C33)(1-β) < θ3(R32-R33).

#### Scenario 3

When the local government, enterprises, and the public jointly choose a coordinated strategy for constructing major railroad projects. When the multi-agent decision jointly selects a coordinated strategy to achieve long-term evolutionary stability of the strategy combination, the pure strategy’s internal equilibrium point E_1_(0,0,0,0,0) is the evolutionally stable equilibrium point. In this case, the Jacobian matrix’s eigenvalue corresponding to the equilibrium point E1 must simultaneously meet the condition of less than 0 so that the system obtains long-term ESE at this point. Three of the three evolution stable strategy systems need to satisfy the conditions (C13-C11) < θ1(R13-R11), (C13-C12) < θ1(R13-R12), (C23-C21)(1 + α) < θ2(R23-R21), (C23-C22)(1 + α) < θ2(R23-R22), (C33-C31)(1-β) < θ3(R33-R31), and (C33-C32)(1-β) < θ3(R33-R32).

Based on the analysis of the evolutionary stability strategy of the asymmetric evolutionary game model for localized emergency response decision-making in major railroad projects, the stability conditions of the tripartite three-strategy cooperative decision-making system at different equilibrium points are discussed. The asymmetric evolutionary game model of cooperative decision-making is simulated with localized emergency response decision combinations in major railroad projects.

## Simulation results with three scenarios

Now, a simulation experiment is carried out to model the selection of emergency response strategies for major railroad construction, and the long-term Nash equilibrium is obtained for the pure strategies’ internal equilibrium point E_20_(1,0,1,0,1,0), E_27_(0,1,0,1, 1), and E_1_(0,0,0,0,0,0). Combined with the Yushu earthquake data from April 10, 2010, the influence indicators under each strategy were fitted, and a long-term Nash equilibrium simulation was carried out for the tripartite collaborative decision-making of the local government, enterprises, and the public.

For strategy combination 1 (the joint selection of economic strategies by the local government, enterprises, and the public), the initial values of x, p, and u are evaluated from 0 to 1 at 0.1 intervals in the three-dimensional decision space [0,1]x[0,1]x[0,1]. A multi-round tripartite repeated evolutionary game dynamic simulation is carried out. The phase trajectory of the tripartite decision strategy combination (x,p,u) in the long-term evolution and development process is observed, and the simulation time t = 10 is used.

Rescue materials, transportation support, and personnel deployment represent major cost components for local governments during emergency responses. According to data from the China Emergency Management Bureau, the Ministry of Public Security urgently mobilized over 1,600 Special Weapons and Tactics (SWAT) and firefighting personnel. It also provided materials valued at more than CNY 1.01 million, including tents and medical supplies, to the affected areas. In addition, the government assumed significant responsibilities in logistical support, such as traffic management and material distribution.

Government-led rescue efforts are typically categorized under the **timeliness strategy**. Therefore, the corresponding cost for the local government under this strategy (C12) is set at 2000, based on resource investment data disclosed by the China Emergency Management Department^42^. The **economic strategy**, which focuses on saving and optimizing resources, incurs a lower cost than C12. This reflects more restrained government actions aimed at minimizing expenditure while maintaining efficiency. The **coordinated strategy** cost (C13) lies between economic and timeliness strategies (C11 and C12). This reflects the involvement of multiple stakeholders and the added costs of cooperation and resource coordination.

Han et al. (2017) provide a theoretical framework for understanding the reputational impacts of emergency response strategies on participating agents, analyzing the decision-making processes of both governments and enterprises^43^. In this model, the parameter R12 is set to a 1700 value based on government data.

The strategy adopted during an emergency directly influences the reputational outcomes for local governments, enterprises, and the public. When local governments adopt **economic strategies**, reputation is enhanced by demonstrating effective resource management, reducing unnecessary expenditure, and optimizing response procedures (R11 = 1100). Under **coordinated strategies**, reputation is improved through visible collaboration across government agencies, enterprises, and social organizations, which enhances cross-sectoral effectiveness (R13 = 1950).

For enterprises, adopting **economic strategies** earns public recognition through efficient service delivery and prudent resource use (R21 = 120). **Timeliness strategies** help enterprises gain trust by ensuring rapid response, thereby improving public and government evaluations (R22 = 135). **Coordinated strategies**, focused on collaboration with the government and social organizations, further increase reputational gains in post-disaster recovery efforts (R23 = 190).

Their role influences the public’s reputation in promoting efficient resource utilization. It improves when emergency resources are distributed rationally and conserved appropriately (R31 = 110). Recognition of rapid government and enterprise responses also contributes positively (R32 = 160). Public approval of joint efforts between government and enterprises further enhances their collective standing (R33 = 130).

Each strategy contributes to post-disaster emergency response outcomes through cost efficiency, response speed, or collaborative effectiveness. These strategies also play a key role in shaping the reputations of all participating agents.

Z_1_ = (C11, C12, C13, C21, C22, C23, C31, C32, C33, α, β, θ1, θ2, θ3, R11, R12, R13, R21, R22, R23, R31, R32, R33) = (1500, 2000, 2200, 150, 160, 180, 130, 170, 140, 0.3, 0.2, 0.7, 0.5, 0.3, 1100, 1700, 1950, 120, 135, 190, 110, 160, 130).

**Fig. 1:**
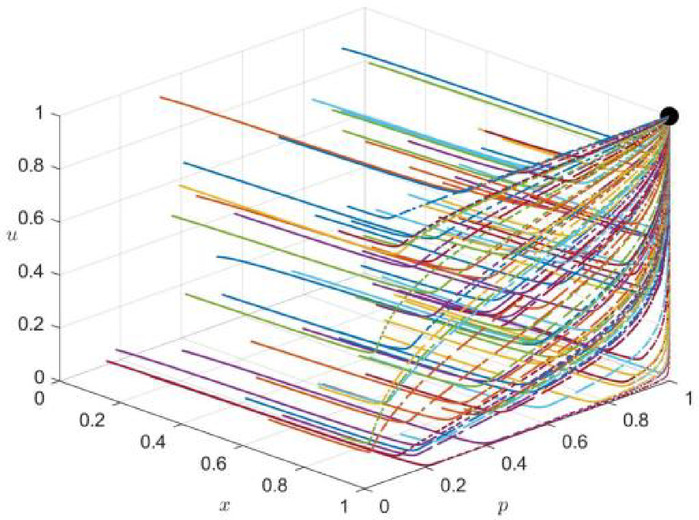
Phase trajectory diagram under strategy combination 1 scenario (x,p,u).

The simulation results are shown in Fig. 1. E_20_ stability during the evolution of internal equilibrium is shown.

For strategy combination 2 (the local government, enterprises, and the public jointly select the timeliness strategy), the initial values of x, p, and u are evaluated from 0 to 1 at 0.1 intervals in the three-dimensional decision space [0,1]x[0,1]x[0,1], and multi-round tripartite repeated evolutionary game dynamic simulation is carried out.

**Fig. 2:**
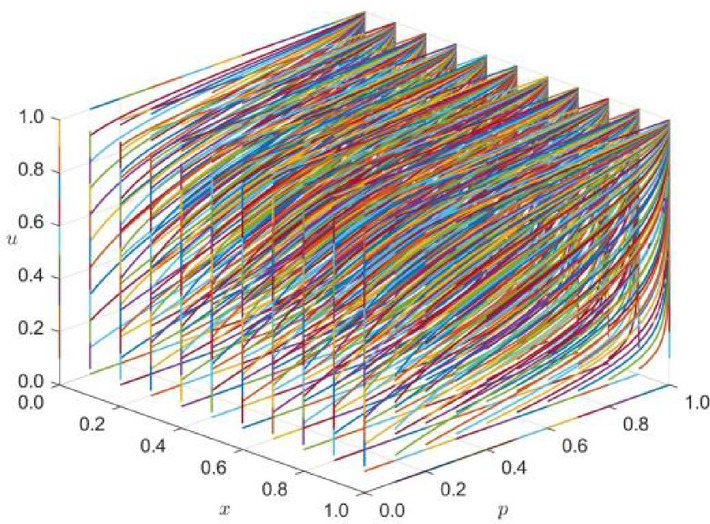
Phase trajectory diagram under the strategy combination 2 scenario (x,p,u).

The phase trajectory of the tripartite decision strategy combination (x,p,u) in the long-term evolution and development process is observed, and the simulation is performed at time t = 10. Z_2_ = (C11, C12, C13, C21, C22, C23, C31, C32, C33, α, β, θ1, θ2, θ3, R11, R12, R13, R21, R22, R23, R31, R32, R33) = (1500, 2000, 2200, 150, 160, 180, 130, 170, 140, 0.3, 0.2, 0.7, 0.5, 0.3, 1100, 1900, 2100, 120, 150, 170, 130, 240, 140). The simulation results are shown in Fig. 2. E_27_ is stable during the evolution of internal equilibrium.

For strategy combination 3 (the local government, enterprises, and the public jointly select the coordinated strategy), the initial values of x, p, and u are evaluated from 0 to 1 at intervals of 0.1 in the three-dimensional decision space [0,1]x[0,1]x[0,1], and multi-round tripartite repeated evolutionary game dynamic simulation is carried out.

**Fig. 3:**
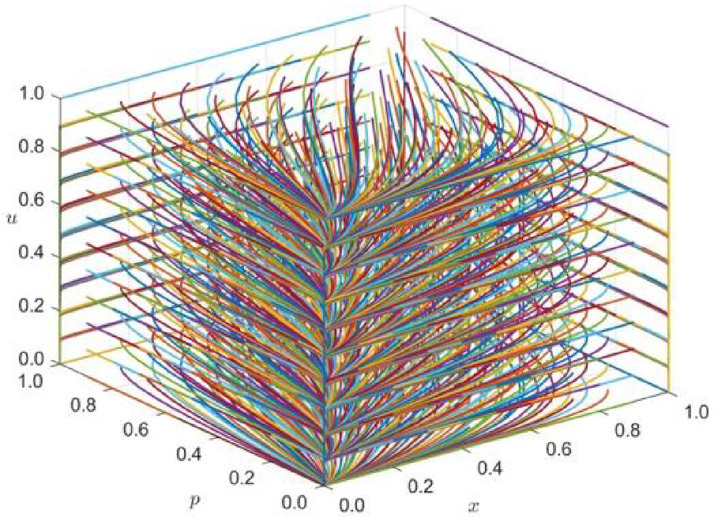
Phase trajectory diagram under the strategy combination 3 scenario (x,p,u).

The phase trajectory of the tripartite decision strategy combination (x,p,u) in the long-term evolution and development process is observed, and the simulation time t = 10 is used. Z_1_ = (C11, C12, C13, C21, C22, C23, C31,C32,C33,α,β,θ1,θ2,θ3,R11,R12,R13,R21,R22,R23,R31,R32,R33) = (1500,2000,2200,150,160,180,130,170,140,0.3,0.2,0.7,0.5,0.3,1100,1900,2200,120,250,320,130,200,160). The simulation results, as shown in Fig. 3, indicate stability in the evolution of the internal equilibrium E_1_.

**Fig. 4:**
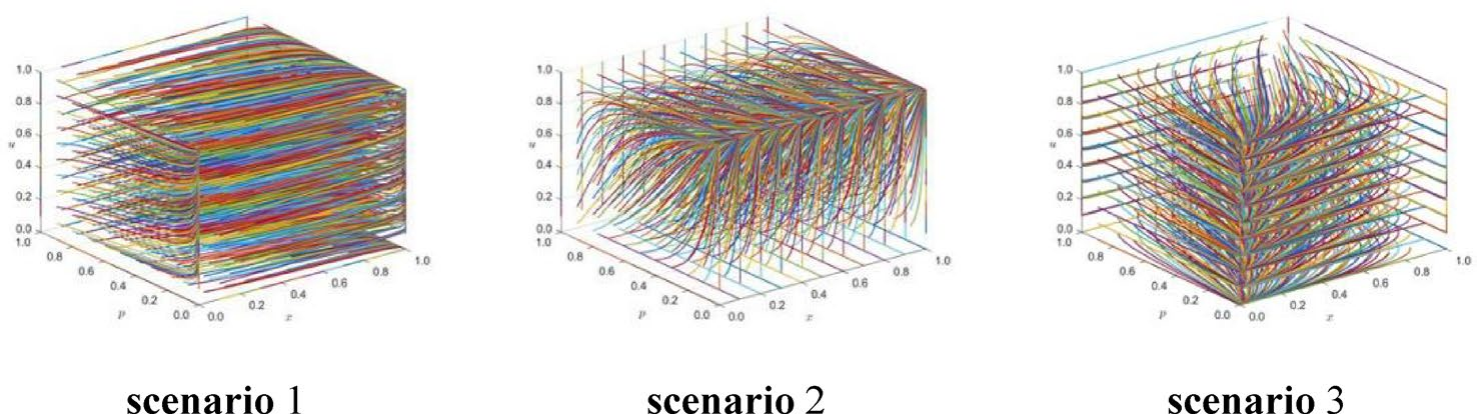
Phase trajectories of the three strategy combinations when the government does not subsidize.

Sensitivity analysis of the relevant parameters shows that, under conditions of constant cost and reputation income, when the local government provides no subsidies for the emergency response actions of enterprises and the public (i.e., α = 0 and β = 0), the phase trajectory diagram of the strategy combination scenario is presented in Fig. 4. Under these conditions, the convergence speed of strategy combinations 1 and 3 changes. However, the equilibrium trend remains stable. In contrast, the equilibrium trend of strategy combination 2 undergoes a significant shift.

**Fig. 5:**
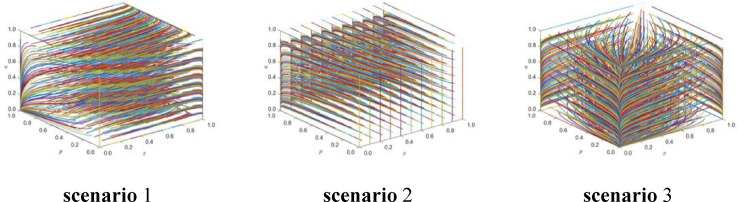
Phase trajectories of the three strategy combinations when fully subsidized by the government.

When the local government provides full subsidies to both enterprises and the public for emergency response activities (i.e., α = 1 and β = 1), the phase trajectory diagram is shown in Fig. 5. Under this scenario, the convergence speed of strategy combinations 2 and 3 increases. Despite this acceleration, the overall equilibrium trends of these strategies remain unchanged. However, strategy combination 1 shows a noticeable change in its equilibrium trend.

These findings indicate that strategy combination 3 remains stable when costs and benefits are fixed. This suggests that it represents a robust option and can serve as a reliable reference for decision-making in emergency rescue management.

## Discussion

Since studies rarely consider the synergistic interactions among the government, enterprises, and the public, this study extends the model framework by incorporating all three actors into a unified game system. This approach enhances the systematic nature and comprehensiveness of emergency rescue decision-making. Compared with previous studies, the model integrates parameters such as emergency rescue cost, reputation effect, subsidy coefficient for emergency rescue, and interest preference coefficient. These parameters reflect different emergency rescue scenarios and broaden both the analytical perspective and the emergency cooperation model’s scope. The model’s assumptions and parameter settings are based on documented railway emergency rescue practices during the Yushu earthquake in China. Nonetheless, the results offer practical reference value for improving railway emergency rescue effectiveness in other national contexts.

Without government subsidies, the tripartite collaborative decision-making system involving three strategies tends to select the economically viable and coordinated option. Under these conditions, all agents focus more on cost control. Enterprises and the public, in particular, prioritize economic considerations and seek to minimize input into emergency responses. However, even without subsidies, public demand and reputation still influence decision-making. The pressure from public opinion continues to encourage all parties to adopt coordinated strategies to gain broader societal support and mitigate potential negative effects.

When the government fully subsidizes emergency rescue operations, the system shifts toward a time-efficient and coordinated strategy. Full subsidies reduce the financial burden on enterprises and the public. Nonetheless, public expectations and reputational concerns continue to shape the decision-making processes of all stakeholders. Under the combined influence of economic relief and public opinion, the focus shifts to rescue timeliness. A prompt and effective response promotes collaboration among the three parties involved.

Recent trends in emergency response show that local governments, enterprises, and the public are playing increasingly valuable roles in major railway projects. As the primary body responsible for emergency management, the local government can influence scenario outcomes by adjusting subsidy policies and reputation incentives. These adjustments affect the strategic mix’s evolution, stability, and equilibrium. Under bounded rationality, the joint selection of coordinated strategies by all three actors can lead to long-term strategic stability. This highlights the importance of balancing interests and reputation to ensure sustained collaboration in multi-agent systems.

At the strategic level, several actions can enhance the efficiency of localized emergency responses in China’s major railway projects. First, the government’s leadership role needs to be reinforced, while enterprises and the public actively participate to form an effective tripartite collaboration mechanism. Implementing digital emergency response platforms, along with regular drills and simulations, can strengthen coordinated rescue capabilities. These measures ensure the proper execution of emergency plans, improve transparency, and enhance public trust and support for rescue operations.

Second, flexible subsidy policies are necessary. The government needs to tailor subsidy mechanisms according to different emergency scenarios. In cases involving full subsidies, governmental support can significantly improve the emergency response speed of all actors. Therefore, a targeted subsidy system for major railway emergencies could be established to guarantee rapid and effective rescue efforts.

Public participation is key in improving the quality and effectiveness of emergency decision-making. It enhances decision quality and helps reduce conflict by enabling the public, stakeholders, experts, and decision-makers to exchange information, express opinions, and engage in mutual learning^44^. Strengthening public engagement through improved communication platforms, community initiatives, and real-time data sharing can enhance both process and outcomes^45^. Public participation also introduces monitoring and pressure, improving decision legitimacy and societal outcomes. Moreover, such participation encourages adaptability and policy responsiveness, promotes a fairer distribution of political power, and strengthens democratic governance^46^. As a form of participation, media-driven public opinion can put further pressure on companies and organizations to implement positive changes^47^.

While important insights are offered into multi-agent collaboration, a key limitation lies in its deterministic modeling approach. The current model does not incorporate stochastic factors such as the probability of secondary disasters (e.g., aftershocks or landslides). These uncertainties can significantly alter resource allocation and influence decision-making in real-world railway emergencies. Future studies should consider integrating dynamic perturbation elements, such as probabilistic models of secondary events, into the evolutionary game framework. This would improve the robustness of simulations under uncertain conditions and provide deeper insights into adaptive strategies and risk management for large-scale railway projects.

Due to limited empirical data, though, the study’s reputation parameters are based on theoretical and logical assumptions. Future empirical research should collect relevant data to improve the accuracy and representativeness of these parameters.

Although the Yushu earthquake provides a valuable case for illustrating collaborative decision-making, the current analysis is geographically and institutionally confined to China. China’s unitary political structure and collectivist culture shape emergency management strategies in distinctive ways. These strategies may not translate directly to countries with different political or cultural frameworks.

Emergency management often involves centralized decision-making and coordination in a unitary state such as the United Kingdom. This system supports rapid policy implementation and harmonized responses, especially during large-scale emergencies. However, such centralization may limit local flexibility and hinder region-specific responses. For example, during the COVID-19 pandemic, the UK’s centralized health system facilitated swift resource deployment, but local authorities encountered difficulties adapting national guidance to local needs^48^.

In contrast, federal systems like those of the United States allow greater regional autonomy in emergency management. This decentralization enables tailored responses considering local risks and resources^49^. However, it can also complicate coordination across jurisdictions, potentially resulting in inconsistent or delayed actions. The response to Hurricane Katrina exemplifies these challenges. Coordination issues between federal, state, and local agencies led to problems in resource allocation and communication, highlighting the importance of strong intergovernmental cooperation^50^.

Cultural differences also influence emergency strategies. In collectivist societies such as China, emergency management emphasizes group welfare and coordinated local government responses. These norms promote rapid mobilization and collective action, as observed during the Yushu earthquake. However, a strong focus on cohesion may limit individual initiative, which is sometimes needed for innovative problem-solving in complex emergencies.

In individualistic societies such as the United States, emergency response emphasizes personal responsibility and initiative. This encourages innovative and decentralized solutions, as individuals and private organizations often play active roles. Crowdfunding and grassroots volunteer initiatives frequently emerge during disasters to fill gaps in official responses. However, excessive reliance on individualism can hinder cohesive government actions and lead to fragmented or inequitable outcomes.

Exploring how collaborative strategies function across diverse political and cultural contexts would provide valuable insights for adapting the proposed model. This broader perspective could support the model’s application on a global scale.

## Conclusion

This paper examines the decision-making processes involved in localized emergency responses for major railroad projects in China, with consideration given to constraints related to resources, urgency, and public needs. An asymmetric EGT model analyzes how economic strategies, timeliness, and coordination affect multi-agent collaboration among local governments, enterprises, and the public. The study identifies the mechanisms and pathways that lead to the formation of evolutionary stable strategies for emergency response organizations across these three stakeholder groups.

The theoretical contributions are threefold. First, it offers insights into multi-agent collaboration by illustrating how economic interests, public demand, and reputational concerns shape collaborative decision-making during emergencies. These findings highlight the importance of balancing diverse interests to achieve effective cooperation. Second, understanding critical strategic elements is enhanced, including economic efficiency, timely response, coordination, and their roles in successful emergency management. These elements are shown to be important for improving overall response outcomes. Third, the theoretical findings support practical recommendations, such as adopting flexible subsidy policies, developing robust collaboration mechanisms, and encouraging stakeholder transparency and trust.

Based on the analysis, the following measures are recommended to improve the effectiveness of emergency response systems for major railroad projects:

1. Policymakers are advised to implement a dynamic subsidy framework guided by emergency assessments. Results indicate that subsidy levels directly influence strategic decisions. Full subsidies are associated with faster responses and greater coordination, while minimal subsidies promote economically conservative strategies. Therefore, a tiered subsidy system needs to be adopted. Emergency severity could be rated on a scale from 1 to 5. Subsidy levels may then be adjusted according to the extent of railway disruption, threat to life and safety, and projected economic losses. For example, a level 1 event may warrant a basic subsidy, whereas a level 5 event could justify maximum financial support. A rapid adjustment mechanism could be established to modify subsidy allocations within 24 h of an incident. A dedicated emergency fund management team needs to be responsible for approval and distribution.
2. An inter-agency railroad emergency response coordination center needs to be established. This center would include an integrated communication platform that enables real-time video conferencing and file sharing among local governments, railway enterprises, and public service organizations (e.g., fire departments and hospitals). Simulation results show that coordinated cross-agency collaboration strategies lead to more stable outcomes and improved reputations. The center could also develop a resource database that inventories emergency equipment – such as recovery vehicles, medical supplies, generators – and personnel from all relevant agencies, including rescue and technical teams. Regular training exercises and the use of standardized coordination tools should enhance efficiency. Such preparations ensure that resources can be deployed rapidly, reducing response times and increasing overall effectiveness during emergencies.

Future research may extend the proposed framework by incorporating stochastic models to address uncertainties, such as secondary disasters. Additional investigation into the influence of contextual variables and collaborative decision-making processes across other engineering domains, such as energy infrastructure or public health emergencies, could yield further insights. Moreover, improving dynamic game models by integrating probability estimates and real-time data would refine emergency response strategies and strengthen the model’s applicability across diverse scenarios.

## Data Availability

The datasets generated and/or analyzed during the current study are available in the Yushu Earthquake Recovery and Reconstruction Master Plan repository; The State Council of the People’s Republic of China, https://www.gov.cn/zwgk/2010-06/13/content_1626853.htm; and General Office of the State Council of the People’s Republic of China, https://www.gov.cn/jrzg/2010-04/14/content_1580651.htm.
